# Topiramate-Induced Bilateral Acute Angle Closure in a Healthy Young Female

**DOI:** 10.7759/cureus.96065

**Published:** 2025-11-04

**Authors:** Michael J Miyashiro, William A Newsom, Nikki P Inamine, Gary S Inamine

**Affiliations:** 1 Ophthalmology, Eyeball Doc LLC, Bradenton, USA; 2 Ophthalmology, Newsom Eye and Laser Center, Gainesville, USA; 3 Internal Medicine, The Queen's Health Systems, Honolulu, USA

**Keywords:** angle closure glaucoma, ciliochoroidal effusion, drug-induced ocular adverse effects, myopic shift, topiramate

## Abstract

Topiramate is a widely prescribed medication for epilepsy, migraine prophylaxis, and weight management, but it can rarely cause acute bilateral angle closure with myopic shift. We describe a healthy 35-year-old woman who developed sudden bilateral painful red eyes, blurred vision, and a myopic shift within one week of initiating topiramate. Examination revealed shallow anterior chambers, elevated intraocular pressure, and iridocorneal touch bilaterally.

Prompt discontinuation of topiramate led to full resolution. Laser peripheral iridotomies (LPIs) were performed while the etiology was uncertain, and are not typically required in medication-induced effusion-related angle closure. This case underscores that cycloplegia and drug cessation are the key interventions, while LPI may be performed only when the underlying mechanism is unclear.

This case highlights the importance of recognizing medication-induced angle closure and distinguishing it from primary pupillary-block mechanisms to ensure timely, vision-saving management.

## Introduction

Topiramate is a sulfamate-substituted monosaccharide that has gained widespread use as an anticonvulsant, migraine prophylactic, and adjunct for weight management [1,2]. Since its approval, topiramate has been associated with rare but serious ophthalmic complications, including bilateral acute angle-closure glaucoma and transient myopic shift described in early case reports [3,4].

Although topiramate has been used off-label for weight management, this is not an FDA-approved indication.

The pathogenesis is distinct from that of primary angle closure. Rather than pupillary block, topiramate induces ciliochoroidal effusion, leading to anterior displacement of the lens-iris diaphragm and secondary shallowing of the anterior chamber [5,6]. This results in sudden myopia, corneal edema, and markedly elevated intraocular pressures, often in patients without traditional glaucoma risk factors. A case series by Senthil et al. also demonstrated that most patients developed symptoms within the first week of therapy initiation, emphasizing the rapid onset of this mechanism [6].

Sulfa-derived compounds such as topiramate can alter fluid dynamics within the choroid and ciliary body by increasing vascular permeability and disrupting osmotic gradients. This promotes ciliochoroidal effusion and anterior rotation of the ciliary body, resulting in forward displacement of the lens-iris diaphragm and a transient myopic shift [5].

Early recognition is crucial, as discontinuation of the drug and initiation of appropriate medical therapy usually result in rapid and complete recovery of ocular anatomy and vision. We present a case of topiramate-induced bilateral acute angle closure in a young, otherwise healthy woman, underscoring the importance of interdisciplinary awareness among neurologists, ophthalmologists, and primary care providers.

## Case presentation

A 35-year-old woman with no prior ocular history presented with bilateral painful red eyes and blurred vision of less than 24 hours’ duration. She had recently initiated topiramate 25 mg daily as monotherapy for weight management, taken for less than one week. Symptoms developed on day 6 after initiation of 25 mg daily topiramate.

**Figure 1:**
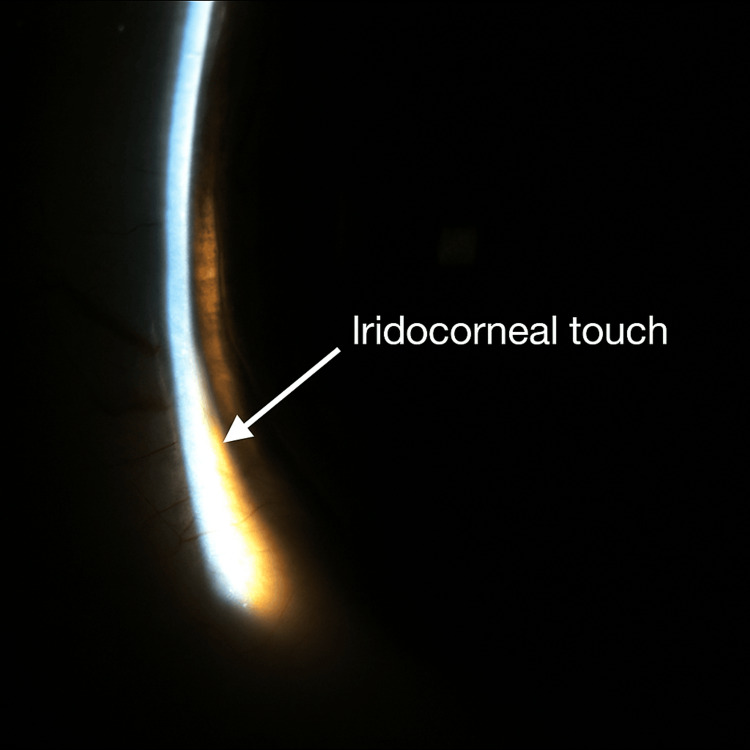
Slit-lamp photograph showing shallow anterior chamber and peripheral iridocorneal touch prior to iridotomy, right eye (white arrow = areas of touch)

**Figure 2:**
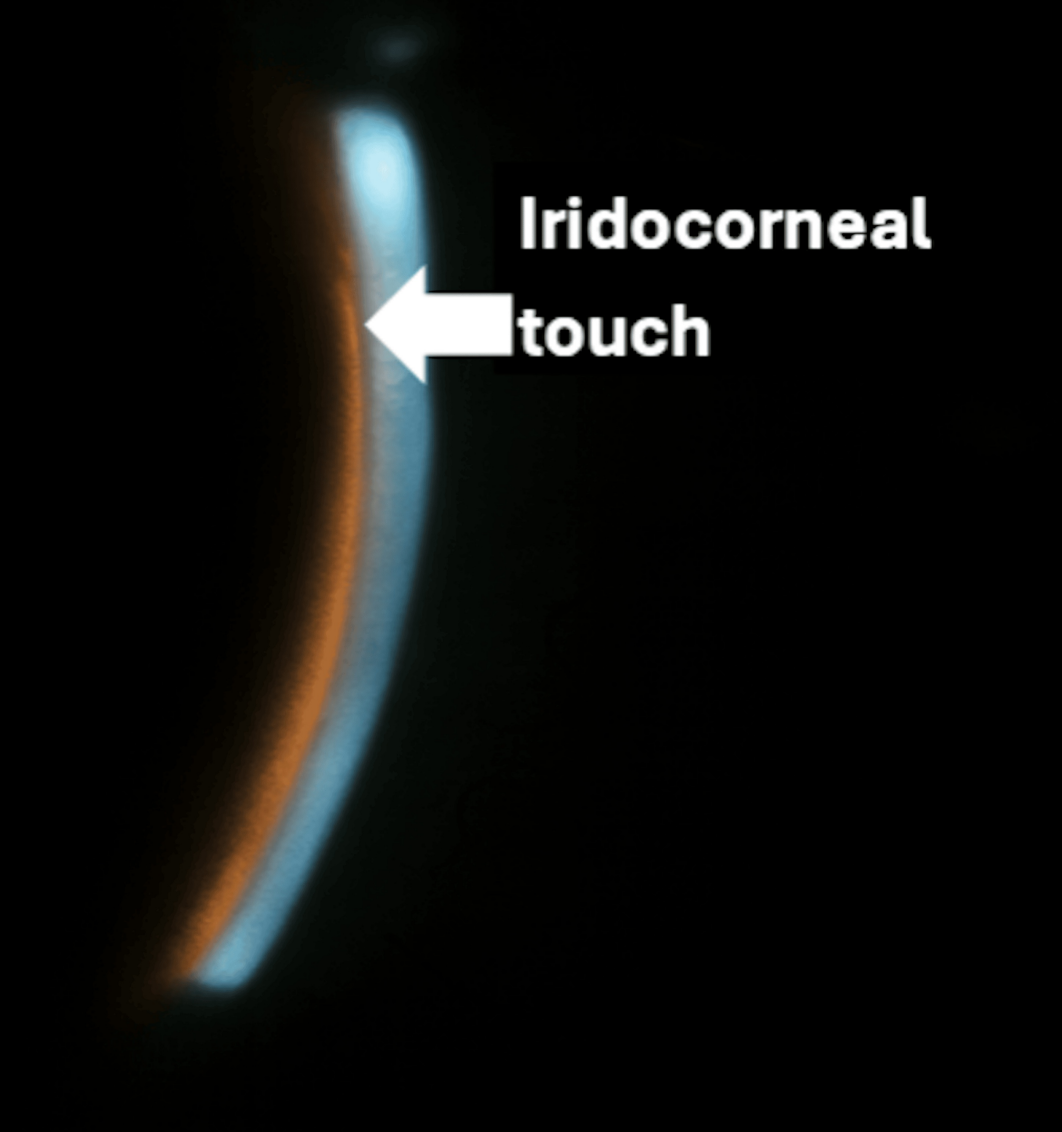
Slit-lamp photograph showing shallow anterior chamber and iridocorneal touch prior to iridotomy, left eye (white arrow = areas of touch)

Examination revealed uncorrected visual acuity of 20/400 in both eyes, with pinholing to 20/25 bilaterally. Manifest refraction demonstrated a myopic shift (right eye −5.00 D, left eye −4.75 D). Anterior segment examination showed +1 conjunctival injection and markedly shallow anterior chambers with iridocorneal touch peripherally in both eyes (Figures 1, 2).

Intraocular pressure measured 26 mm Hg bilaterally by Goldmann applanation. Fundus examination was unremarkable. The diagnosis of topiramate-induced bilateral angle closure was made.

**Figure 3:**
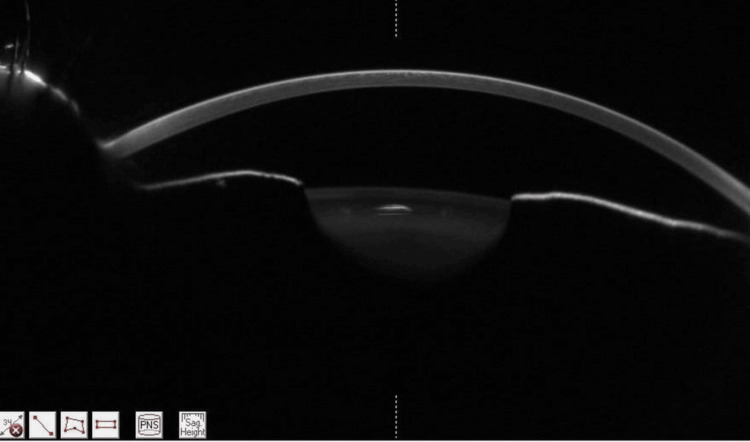
Pentacam HR (Oculus Inc., Wetzlar, Germany) showing deepened anterior chamber post-LPI and topiramate discontinuation ACD 3.1 mm, AC volume 150 mm³. LPI, laser peripheral iridotomy; ACD, anterior chamber depth; AC, anterior chamber.

**Figure 4:**
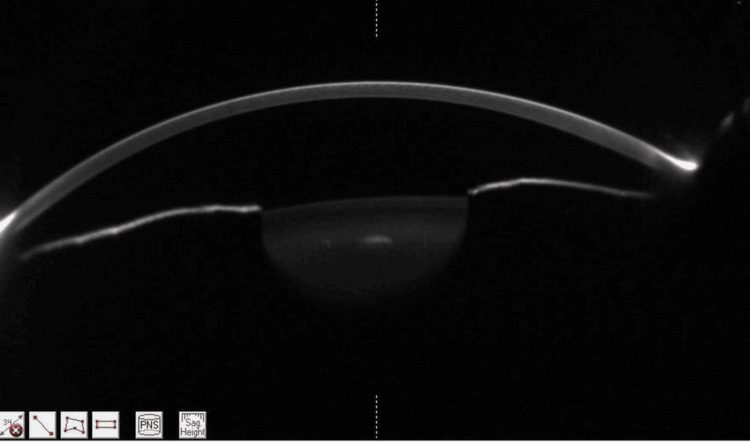
Pentacam HR (Oculus Inc., Wetzlar, Germany) left eye showing restored anterior chamber depth post-LPI and drug cessation ACD 3.1 mm, AC volume 150 mm³. LPI, laser peripheral iridotomy; ACD, anterior chamber depth; AC, anterior chamber.

Topiramate was discontinued immediately. Laser peripheral iridotomy (LPI) was performed in the right eye on the day of presentation, followed by the left eye the next day (Figures 3, 4).

Pilocarpine was used only peri-procedurally to facilitate iridotomy formation and was not continued thereafter.

Peripheral iridotomies were successfully created at the 11 o’clock position in both eyes without complication. Prednisolone acetate 1% was initiated post-procedure. No topical or systemic anti-glaucoma agents were used, as intraocular pressures normalized promptly after drug cessation and iridotomy. Within one week, both eyes demonstrated deep anterior chambers, patent iridotomies, intraocular pressures of 15 mm Hg bilaterally, and uncorrected visual acuity of 20/20 (Table 1).

**Table 1:** Timeline of clinical course IOP, intraocular pressure; ACD, anterior chamber depth; AC, anterior chamber.

Time point	Visual acuity	Refraction	IOP (mm Hg)	Shaffer grade	ACD (mm)	AC volume (mm³)	AC angle (°)	Notes
Presentation	20/400 (20/25 pinhole)	−5.00/−4.75 D	26/26	0-1	1.7	60	12	Bilateral iridocorneal touch; ciliochoroidal effusion presumed
24-48 h	20/60 → 20/30	−2.00 D	20/18	2-3	2.4	95	22	Pressures improving after drug cessation; chamber deepening
1 week	20/20	Plano	15/15	4	3.1	150	36	Angles fully open; anatomy normalized

## Discussion

Topiramate-induced bilateral angle closure has become increasingly recognized since the first reports in the early 2000s [1,2]. Unlike primary angle closure, which typically affects older patients with anatomically narrow angles, this condition arises in younger, otherwise healthy individuals and is bilateral in most cases [3,5]. Female predominance has been noted, consistent with the demographics of migraine prophylaxis therapy [2,4].

The underlying mechanism differs from pupillary block. Topiramate induces ciliochoroidal effusion, which leads to anterior rotation of the ciliary body and forward displacement of the lens-iris diaphragm. This results in acute myopia, shallowing of the anterior chamber, and secondary angle closure [5,6]. Imaging modalities such as ultrasound biomicroscopy and anterior segment optical coherence tomography have confirmed these anatomic changes, showing ciliary body edema, supraciliary fluid, and appositional angle closure [7]. These findings help distinguish topiramate-induced angle closure from primary mechanisms.

Baseline AS-OCT (anterior segment optical coherence tomography) or UBM (ultrasound biomicroscopy) imaging was not obtained at presentation due to corneal edema and patient discomfort; however, post-resolution Pentacam cross-sections confirmed anatomic normalization consistent with the effusion-resolution mechanism.

The onset of symptoms is usually within the first two weeks of therapy initiation or dose escalation [2,5]. A case series from India reported that most patients developed symptoms within one week of starting topiramate, often with intraocular pressures exceeding 40 mm Hg [7]. Clinically, patients present with sudden blurred vision, myopic shift, ocular pain, headache, and halos around lights. Because the presentation can mimic uveitis, conjunctivitis, or nonspecific migraine symptoms, delays in ophthalmic referral are common [4,6].

Management requires a high index of suspicion. Immediate discontinuation of topiramate is the most important step, as ongoing use perpetuates ciliochoroidal swelling [1,2,5]. Cycloplegic agents help reverse anterior displacement of the lens-iris diaphragm, and aqueous suppressants can aid in lowering intraocular pressure. Hyperosmotic agents may be required in more severe cases. Importantly, pilocarpine and LPI are generally ineffective and may exacerbate forward rotation of the ciliary body [3,6]; therefore, miotic agents are typically avoided in topiramate-induced angle closure. In this case, pilocarpine was applied briefly in a peri-procedural setting to facilitate laser iridotomy before the effusion mechanism was fully recognized - a real-world reflection of early diagnostic uncertainty.

In this case, LPI was performed early as a diagnostic and precautionary measure before the effusion mechanism was fully recognized. Although generally not curative in medication-induced cases, it may still be performed when the etiology is initially uncertain [3,6]. Prognosis is favorable if the condition is recognized promptly. Most patients regain their baseline vision and angle anatomy within days to weeks following cessation of the drug and appropriate management [2,6,7]. However, optic nerve damage and permanent glaucomatous changes have been reported in delayed diagnoses. These risks highlight the importance of interdisciplinary communication between prescribing physicians and ophthalmologists. Neurologists, psychiatrists, and primary care providers should be aware of this rare but potentially vision-threatening adverse event [4,6].

## Conclusions

Topiramate-induced bilateral acute angle closure is an uncommon but clinically significant condition that requires a high index of suspicion for timely recognition. Even low-dose monotherapy can precipitate this reaction in otherwise healthy individuals. Prompt discontinuation of the medication and use of cycloplegic agents are the cornerstones of management, effectively reversing anterior displacement of the lens-iris diaphragm. Procedures such as LPI may be performed when the diagnosis is uncertain or to exclude concurrent pupillary block, but they are not typically required for recovery. This case highlights the importance of differentiating drug-induced secondary mechanisms from primary angle closure to avoid unnecessary interventions and preserve vision. Greater awareness among neurologists, psychiatrists, ophthalmologists, and primary care physicians is essential to ensure early identification of this reversible cause of bilateral angle closure. Reporting such cases contributes to improved pharmacovigilance and a broader understanding of medication-related ocular emergencies.
